# RhoA/ROCK/GSK3β Signaling: A Keystone in Understanding Alzheimer’s Disease

**DOI:** 10.3390/cimb47020124

**Published:** 2025-02-14

**Authors:** Milan M. Medd, Jayden E. Yon, Hongxin Dong

**Affiliations:** 1 Department of Urology, Feinberg School of Medicine, Northwestern University, Chicago, IL 60611, USA; milanmedd@canzxbio.com (M.M.M.); yonjayden410@gmail.com (J.E.Y.); 2 Stephen M. Stahl Center for Psychiatric Neuroscience, Departments of Psychiatry & Behavioral Sciences and Neurology, Feinberg School of Medicine, Northwestern University, Chicago, IL 60611, USA

**Keywords:** GSK3β, RhoA, ROCK, Alzheimer’s disease, sTREM2, transgelin-2

## Abstract

Alzheimer’s disease (AD) is a neurodegenerative disease characterized by progressive cognitive decline and loss of neuronal integrity. Emerging evidence suggests that RhoA, Rho-associated coiled-coil kinase (ROCK), and their downstream effector molecule glycogen synthase 3β (GSK3β) interact within a complex signaling pathway (RhoA/ROCK/GSK3β) that plays a crucial role in the pathogenesis of AD. RhoA, a small GTPase, along with its downstream effector, ROCK, regulates various cellular processes, including actin cytoskeleton dynamics, apoptosis, and synaptic plasticity. GSK3β, a serine/threonine kinase, plays a key role in neuronal function and AD pathology, including the regulation of tau phosphorylation and amyloid-beta cleavage. Overactive GSK3β has been closely linked to tau hyperphosphorylation, neurodegeneration, and the progression of AD. Thus, GSK3β has been considered as a promising therapeutic target for treating AD and mitigating cognitive impairment. However, clinical trials of GSK3β in AD have faced considerable challenges due to the complexity of the specific neuronal inhibition of GSK3β. In this review, we summarize the literature regarding the relationship of RhoA/ROCK and GSK3β signaling pathways in AD pathogenesis. We further discuss recent findings of the sTREM2-transgelin-2 (TG2) axis as a potential mediator of this complex pathway and provide our review on a novel targeting strategy for AD.

## 1. Introduction

Alzheimer’s disease (AD) is a progressive neurodegenerative disease characterized by the accumulation of amyloid-beta (Aβ) peptides and neurofibrillary tangles (NFTs) induced by the hyperphosphorylation of tau proteins [1]. AD is the most common form of dementia and constitutes 60–70% of cases of senile dementia [2]. Prevalence of AD increases with age, and current treatment is mostly ineffective in preventing disease progression [3]. The etiology and pathogenesis of AD is complex and is dependent on interactions between cell types in the central nervous system (CNS) and multifaceted signaling pathways that lead to tau hyperphosphorylation [4,5,6,7,8]. Inhibiting GSK3β to activate the Wnt/β-catenin pathway has been proposed as a promising therapeutic avenue and is currently under many active investigations; however, targeting the GSK3β pathway for AD has hitherto presented challenges in clinical settings due to small-molecule inhibitor specificity [8]. In parallel, the Ras homolog family member A (RhoA)/Rho-associated coiled-coil containing protein kinase (ROCK) has emerged as a significant regulator of AD and has also been proposed as a promising therapeutic avenue, though with an unclear definition of the mechanisms involved [9]. In this review, we summarize the to-date investigations of each of these two major pathways and present our view on the potential complex cross-talk between RhoA/ROCK and GSK3β pathways to regulate tau phosphorylation and current knowledge gaps. Signaling by soluble Triggering Receptor Expressed on Myeloid Cells 2 (sTREM2) and its ligand transgelin-2 (TG2) is a newly described pathway that plays a critical role in AD pathogenesis [9]. We also present our view on how the sTREM2/TG2 axis may modulate RhoA/ROCK and GSK3β in regulating tau phosphorylation and serve as a viable therapeutic target.

## 2. RhoA/ROCK Pathway in Neuronal Regulation

RhoA is a small GTPase protein in the Rho family of GTPases composed of three isoforms, RhoA, RhoB, and RhoC [10]. RhoA transduces signals and mediates various cellular processes, including cell migration, gene expression, and vesicle trafficking [11,12,13]. RhoA is abundant in smooth muscle cells, neurons, and immune cells [14,15,16]. RhoA regulates various cellular activities, including cytoskeleton modulation, cell death, mitochondrial homeostasis, autophagy, inflammation, and gene transcription [17]. In the brain, RhoA plays a role in regulating neuronal development, synaptic plasticity, and the progression of neurodegenerative diseases [15,17,18]. The RhoA/ROCK pathway specifically inhibits many of these processes [19,20]. Following brain injury, RhoA has been found to be upregulated and activated, resulting in growth cone collapse and failed axon regeneration [21,22].

RhoA activity is controlled by four main regulatory proteins: guanine nucleotide exchange factors (GEFs), GTPase activating proteins (GAPs), guanine nucleotide dissociation inhibitors (GDIs), and GDI dissociation factors (GDFs) [23]. Over 70 distinct GEFs for Rho GTPases have been identified in mammals [24]. GEFs act as positive regulators of RhoA by exchanging GDP for GTP and activate RhoA by dissociating GDP from the GDP-RhoA complex; this unbound RhoA is subsequently able to bind to GTP [24,25]. While around 80 distinct RhoGAPs have been found in mammals, only a small number of GAPs have been shown to be specific for certain GTPases within the Rho family [23]. RhoGAPs specific for RhoA, but not RhoB or RhoC, have not yet been discovered [23,24]. GAPs act as negative regulators of RhoA by increasing the intrinsic rate of GTP hydrolysis in GTP-bound RhoA [26]. The mechanism by which the intrinsic rate of GTP hydrolysis varies across the diverse pool of GAPs, but RhoGAPs specifically have been found to contain a characteristic RhoGAP domain capable of binding to Rho GTPases and promoting GTP hydrolysis [24,25].

Unlike RhoGEFs and RhoGAPs, only three isoforms of RhoGDIs have been found in mammals [27]. GDIs act as negative regulators of RhoA by inhibiting the dissociation of guanine nucleotides (i.e., GDP) and preventing the loading of GTP to the GDP-RhoA complex [28]. However, they do not inhibit the loading of GDP/GTP to nucleotide-free RhoA [29,30]. RhoGDIs were also found to inhibit RhoA activity, preventing intrinsic and GAP-stimulated GTP hydrolysis [31]. Another function of RhoGDIs is as a chaperone for inactive RhoA. GDIs inhibit GTPase activation in cytosol, where 90–95% of Rho proteins reside in the cell [32,33]. This chaperone function rapidly deploys the inactived RhoA to the cell membrane for activation in response to cellular signals [28]. Additionally, GDFs act as positive regulatory proteins for RhoA and have been proposed to catalyze the dissociation of RhoGTPase-GDI complexes, allowing for activation of Rho GTPases [27,34].

In addition to the various types of Rho GTPase regulatory proteins, post-translational modifications (PTMs) can regulate RhoA activity, such as prenylation and phosphorylation [17]. RhoA activation requires prenylation, as activated RhoA must be membrane-bound [35]. Prenylation of RhoA causes increased hydrophobicity and facilitates membrane association [36]. Alternatively, phosphorylation of RhoA occurs most commonly via protein kinase A (PKA) and cyclic GMP-dependent protein kinase (PKG), both mediating the phosphorylation of Ser188, a residue close to the prenylated cysteine residue of the C-terminal essential for RhoA membrane binding [37,38,39]. The phosphorylation negatively regulates RhoA activity by preventing dissociation of GDP-bound RhoA from RhoGDIs [40]. Ser188 phosphorylation has also been suggested to inhibit the binding of RhoA to ROCK, effectively inhibiting RhoA/ROCK signaling [41].

**Figure 1:**
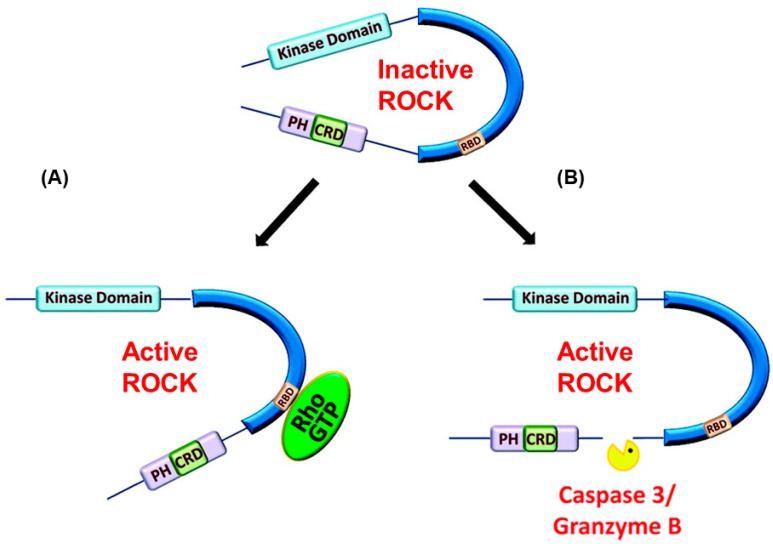
Activation pathways of ROCK. (**A**) Rho GTPase-dependent activation of ROCK through binding to RBD. (**B**) Rho GTPase-independent activation of ROCK through Caspase 3 or Granzyme B cleavage of carboxyl terminal region of ROCK. Images (**A**,**B**) were adapted from [52].

ROCK is a key regulator of the cytoskeleton and impacts various cellular functions, such as cell shape, motility, proliferation, and gene expression [42]. ROCK has two isoforms: ROCK-I and ROCK-II [43]. ROCK-I is prominently expressed in non-neuronal tissues such as the liver, testis, and kidney. ROCK-II is mostly expressed in the brain and skeletal muscle [43]. ROCK is constituted of an N-terminally located kinase domain, coiled-coil domain, and a Rho-binding domain (RBD). The switch regions of activated RhoA bind to the RBD to activate ROCK [44]. At the C-terminus, a pleckstrin homology (PH) domain contains a cysteine-rich C1 domain [45]. The PH-C1 tandem inhibits ROCK by sequestering its N-terminal kinase domain and reducing its kinase activity. The tandem also facilitates binding to membrane bilayers [46]. ROCK is the most widely studied downstream effector molecule of RhoA and is activated when bound to GTP-bound RhoA [47,48]. RhoA activates ROCK through derepression where the PH-C1 tandem-induced autoinhibition of the kinase domain is relieved, activating the kinase domain [49]. Other mechanisms of ROCK activation have been alleviated, such as the binding of arachidonic acid to the PH domain or cleavage of the carboxyl-terminus by granzyme B or caspase-2/3 [50,51] (Figure 1).

RhoA/ROCK signaling has been linked to AD risk factors, mainly tau hyperphosphorylation, synaptic damage, Aβ aggregation, and neuroinflammation [48]. When activated, RhoA/ROCK signaling pathways phosphorylate and activate downstream effectors that are also involved in regulating neuronal processes such as axonal guidance and regeneration and dendritic spine formation [53]. RhoA/ROCK phosphorylates various substrates, including Lim kinase (LMK), myosin light chain (MLC), and collapsing response mediator protein-2 (CRMP-2), which inhibit axonal growth [54]. Activated ROCK also stimulates actomyosin contractility by decreasing myosin phosphatase activity, MLC2 phosphorylation, and resulting in spine shortening and retraction [55]. ROCK-I and ROCK-II differentially regulate actomyosin organization in influencing synaptic polarity [56]. ROCK-I is thought to be responsible for actin cytoskeleton destabilizing through regulating MLC2 phosphorylation while ROCK-II is required for stabilizing the actin cytoskeleton by regulating cofilin phosphorylation [57]. The RhoA/ROCK pathway indirectly activates GSK3β by deactivating GSK3β-inhibitory kinases such as Protein Kinase B (PKB or AKT) [58]. Both ROCK-I and ROCK-II have been shown to regulate RhoA/ROCK signaling to indirectly activate GSK3β. It was shown that the inhibition of either ROCK-I or ROCK-II efficiently inactivated GSK3β and prevented subsequent tau phosphorylation [59,60]. The role of GSK3β in AD will be discussed extensively in the following sections.

## 3. GSK3β Regulation

GSK3β activation is well known to impact AD pathogenesis by regulating tau phosphorylation [61]. GSK-3 has two isoforms in mammals, GSK3α and GSK3β, which share 95% homology in the kinase domain but differ at the N and C terminal regions [62]. GSK3α has slightly longer N and C termini [62]. While both isoforms are ubiquitously expressed, GSK3β has greater abundance in the brain and has specificity for a broad range of substrates, including metabolic proteins, transcription and translation factors, and cytoskeletal proteins [63,64]. GSK3β is abundantly expressed in neurons and plays essential roles in regulating neuronal development, synaptic pruning, and influencing the pathogenesis of neurodegenerative diseases, primarily AD [65]. It was observed that ventricular progenitors in the developing neocortex experience a higher expression of GSK3β that gradually declines following neuronal differentiation [66,67]. Transcripts of GSK3β have two alternative splicing forms, the short form GSK3β1 and the long form GSK3β2. GSK3β2 is neuron-specific, with a high expression during brain development that persists until adulthood. Interestingly, GSK3β2 shows less phosphorylation activity of tau at the AD-associated Ser396 epitope in comparison to GSK3β1 [68], suggesting that GSK3β2 may not be associated with AD. In addition to neurons, *GSK3β* is also expressed in microglia, astrocytes, and oligodendrocytes [69,70,71].

The function of GSK3β is largely mediated by its activation and inhibition through phosphorylation at the Tyr216 and Ser9 residues, respectively [58,72]. Protein kinase B (PKB/Akt), located downstream of phosphatidylinositol 3-kinase (PI3K), has been shown to rapidly phosphorylate Ser9 of GSK3β both in vivo and in vitro with growth factor stimulation [58,73]. cAMP-dependent protein kinase A (PKA) and PKC have also been suggested to phosphorylate Ser9 in vivo and in vitro [74,75]. Phosphorylation of GSK3β at Tyr216 at the activating loop is thought to occur by autophosphorylation, although phosphorylation by other kinases, such as the Src family kinases (SFKs) and Janus kinases (JAKs), has also been reported [76,77,78]. Further, GSK3β can be activated through the dephosphorylation of Ser9 by phosphatases such as protein phosphatase 2 (PP2A) [79]. To date, how GSK3β activity is regulated is not fully understood and remains an active area of investigation.

Dopamine and glutamate are neurotransmitters that have been associated with the hyperactive state of *GSK3β.* A study by Beaulieu et al. observed increased dopaminergic neurotransmission in dopamine transporter null (DAT^−/−^) mice that resulted in reduced PKB activation and subsequent GSK3β hyperactivation [80]. In the presence of glutamate, the interaction between GSK3*β* and glutamatergic *N*-methyl-D-aspartate (NMDA) receptors is believed to be dual-directional [81]. One study reported rapid dephosphorylation of the inhibitory Ser9 residue in GSK3β and simultaneous neurotoxicity in cultured hippocampal neurons following increased NMDA signaling [82]. This suggests that NMDA receptor signaling results in activated GSK3β. NMDA receptor antagonists such as phencyclidine and memantine have also been shown to increase Ser9 phosphorylation of GSK3β in murine models [83,84]. It was later observed that NMDA signaling activates protein phosphatase 1 (PP1), which is capable of dephosphorylating the Ser9 residue [85]. Conversely, GSK3β plays a role in the regulation of NMDA receptor surface localization and function [86]. One of the most well-known clinical inhibitors of GSK3β is lithium [87]. Its mechanism of action encompasses both direct and indirect pathways. Directly, lithium competes with Mg^2+^ ions, cofactors of GSK3β that stabilize the enzyme’s active site, thereby inhibiting its function [88]. Indirectly, lithium enhances the phosphorylation of GSK3β at the Ser9, although the exact reported mechanisms behind lithium-induced inhibition are conflicting and unclear [89,90,91]. Consistent with this notion, chronic lithium exposure has been shown to diminish NMDA receptor signaling and subsequent glutamate-induced excitotoxicity in murine cortical neurons [92,93]. These interactions highlight how GSK3β plays a fundamental role in the body’s response to the usage of psychoactive drugs.

The expression of GSK3β may be regulated epigenetically. The promoter region of GSK3β was shown to contain the *cis*-regulatory molecule (CRM) that binds to cell-type specific transcription factors, such as Sox2, Sox9, and Neurogenin2 (Ngn2) [94]. Using Histone ChIP-seq analysis of the GSK3β genomic region, it was shown that two histone marks, H3K4me3 and H3K27ac, exhibited open chromatin around the promoter region and exon 1 of murine GSK3β [94]. These findings are consistent with prior studies demonstrating that the GSK3β promoter region contains CCAAT/enhancer-binding proteins (C/EBP) consensus sequences, the known binding sites for transcription factors Sox2, Sox9, and Ngn2 [94,95]. It is noteworthy that the *GSK3β* gene is highly conserved evolutionarily across cell types in all eukaryotic species and that *GSK3β* deletion was shown to cause postnatal fatality with multiple developmental defects [96,97]. These studies underscore the essential role of *GSK3β* in both physiological functions and disease pathogenesis.

## 4. *GSK3β* in Neuronal Development

GSK3β is broadly expressed throughout normal tissues and plays a role in numerous cellular functions including gene expression, stress responses, cell survival and death, cell structure, migration, metabolism, and differentiation [63,98,99]. When concentrated to the CNS, GSK3β is essential in regulating neurogenesis, neuronal differentiation, neuronal polarization, and progenitor proliferation [100,101]. It was shown that the silencing of GSK3 in mice resulted in the hyperproliferation of progenitor cells but a suppression of neuronal differentiation, with increased cortical surface area and a thinner cortex [100,102]. It is believed that the ability of GSK3β to mediate neurogenesis, progenitor proliferation, and neuronal differentiation originates from its ability to interact with and regulate various signaling pathways including Wnt, Sonic Hedgehog (SHH), and Notch. These pathways, through interactions with GSK3β, promote the proliferation of neuronal progenitors by either inhibiting GSK3β activity or utilizing GSK3β as a regulatory molecule [103,104,105,106]. In regulating the Wnt pathway, GSK3β exhibits a dual role. Under canonical Wnt ligation, GSK3β can be inhibited by Dishevelled (Dsh), a transducer in the signaling cascade that inhibits GSK3β-mediated phosphorylation and degradation of β-catenin [107,108]. In the absence of Wnt signaling, GSK3β is able to mark β-catenin for proteasomal degradation, thereby preventing progenitor proliferation and differentiation [109]. Similarly, it has been suggested that GSK3β can function as either a positive or a negative regulator of Notch signaling [110,111,112], suggesting the cell type-specific role of GSK3β. It is understood that GSK3β primarily acts as a downstream effector in the SHH pathway, although its role as either a positive or a negative regulator remains similarly controversial [113,114]. Nonetheless, inactivation of GSK3β by phosphorylation at Ser9 by PI3K/AKT has been shown to promote axon formation [115]. Further studies in hippocampal neurons revealed high concentrations of inactive GSK3β at the tips of newly formed axons, in correlation with the proper localization and functioning of Partitioning-Defective 3 (PAR3), a molecule that facilitates neuronal polarity and axon specification [116,117,118]. The dual role of GSK3β in neuronal regulation underscores the complexity of the molecule in neural development and a potential challenge in inhibiting GSK3β for treating AD.

## 5. RhoA/ROCK/GSK3β in Alzheimer’s Disease

**Figure 2:**
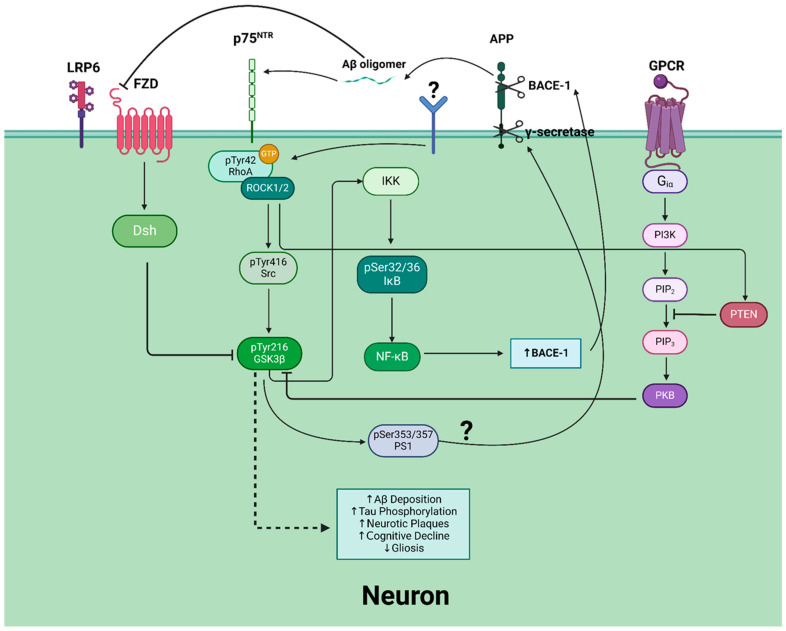
Signaling pathways directly and indirectly linked to RhoA/ROCK-mediated activation of GSK3β and the positive feedback loop between Aβ deposition and GSK3β activation. (1) ROCK phosphorylates and activates PTEN, a phosphatase that inhibits the PI3K/AKT signaling pathway. PTEN dephosphorylates PIP_3_ into PIP_2_, preventing the activation of PKB (AKT). PKB inhibits GSK3β by Ser9 phosphorylation. The inhibition of PKB by RhoA/ROCK activates GSK3β; (2) Aβ ligation of p75^NTR^ activates RhoA by Tyr42 phosphorylation. Activated RhoA/ROCK phosphorylates and activates Src at the Tyr416 residue. Activated Src phosphorylates and activates GSK3β at the Tyr216 residue, subsequently activating IKK. IKK phosphorylates IκB at Ser32/36, marking it for proteasomal degradation and impeding NF-κB inhibition. NF-κB increases BACE-1 expression, which increases the activity of amyloidogenic processing of APP into Aβ. Newly processed Aβ oligomers bind to p75^NTR^ and complete the positive feedback cycle; (3) Aβ binds to the extracellular domains of FZD and LRP6, preventing Wnt ligand interactions with the LRP6/FZD complex and the successive inhibition of GSK3β. It is noteworthy that GSK3β is capable of phosphorylating PS1 and the Ser353 and 357 residues. However, the exact effect on Aβ processing is unclear; (4) overactivation of GSK3β induces AD pathogenesis such as Aβ deposition, tau hyperphosphorylation, neurotic plaque formation, and cognitive decline.

The RhoA/ROCK pathway, when dysregulated, is a recognized significant contributor to tau phosphorylation and subsequent AD development [119]. Emerging evidence suggests that RhoA/ROCK indirectly activates GSK3β through reducing the activity of the inhibitory kinase PKB in the PI3K/AKT pathway (Figure 2). RhoA/ROCK has been found to be able to directly phosphorylate and activate phosphatase and tensin homolog (PTEN) [120,121]. Active PTEN inhibits PI3K signaling by dephosphorylating the lipid signaling intermediate phosphatidylinositol (3,4,5)-triphosphate (PIP_3_) into PIP_2_, effectively impeding the activation of the downstream GSK3β inhibitor PKB [122,123]. Corroboratively, studies have shown that the inhibition of RhoA/ROCK leads to rapid activation of the PI3K/PKB signaling pathway [124]. Whether RhoA/ROCK is able to indirectly inhibit other GSK3β-inhibiting kinases, such as PKA and PKC, remains to be elucidated.

Upon RhoA/ROCK dysregulation, the hyperactivation of GSK3β has been well demonstrated to be a major contributor to the pathogenesis of AD, largely by phosphorylating tubulin-associated units (tau) [125,126,127]. Tau is an intrinsically disordered, microtubule-associated protein predominantly expressed in neurons [128,129]. In its normal state, tau binds to and stabilizes distal axonal microtubules (MTs) [130]. The stabilizing function of tau promotes the polymerization of axonal MTs, an essential regulatory mechanism for proper axonal transport [131,132]. Tau is the substrate of many Ser/Thr kinases, including GSK3β, Cdk5, MARK, PKA, CamKII, MAPK, PKC, JNK, and ROCK [133]. Tau is also the substrate of the tyrosine kinases Fyn and Abl [134]. Phosphorylation of tau at specific residues, most notably Ser202, Ser396, Ser404, Thr181, and Thr231, is thought to induce conformational changes that create a positive feedback loop where tau is hyperphosphorylated via sequential phosphorylation at multiple sites, although the exact relationship between these residues and AD requires further defining [135,136,137,138]. Hyperphosphorylated tau exhibits a reduced affinity for MTs, which results in axonal destabilization [139]. Hyperphosphorylated tau presents aggregation-prone properties and forms neurofibrillary tangles (NFTs), which are a hallmark of AD [140,141].

While RhoA/ROCK is a known activator of neuronal GSK3β, other signaling pathways, such as Cdk5, ERK, and JNK are also key players in AD pathogenesis [133]. Cyclin-dependent kinase 5 (Cdk5) closely modulates Aβ deposition and, when hyperactivated by p25, induces aberrant Aβ cleavage and tau hyperphosphorylation [142,143]. Pharmacological inhibition of Cdk5 activation has been shown to reduce tau phosphorylation and Aβ processing and ameliorates neuronal death in p25 transgenic mice [144,145]. Mitogen-activated protein kinase (MAPK) pathways, including the extracellular signal-regulated kinase 1/2 (ERK), c- Jun N-terminal kinase (JNK), and P38 kinase (P38) pathways, have been shown to influence AD development and progression [146,147]. In AD mouse models, ERK, JNK, and P38 were all found in an overactivated state in the nervous system, spine, and brain respectively [148,149,150]. While moderate ERK activation is necessary for synaptic plasticity, hyperactivated ERK has been associated with NFT formation and early-stage AD-related protein deposition, leading to impaired hippocampal function in AD patients and murine models [146,150,151,152]. Similarly, overactivated P38 has been shown to increase tau phosphorylation and favor amyloidogenic processing of the amyloid precursor protein (APP), while inhibition of JNK by D-JNKI1 is suggested to suppress synaptic shrinkage in AD patients [149,151,152]. Inhibitors of MAPK pathways can reduce Aβ deposition, neuronal apoptosis, memory impairment, and tau hyperphosphorylation, making them a favorable therapeutic target to be investigated [153,154,155].

Activated GSK3β was shown to phosphorylate tau at most of the Ser/Thr residues associated with tau hyperphosphorylation and AD pathogenesis [156,157]. Corroboratively, GSK3β inhibition in diverse mouse models has been shown to reduce tau phosphorylation and improve cognitive impairments measured through behavioral assays [158,159]. By contrast, the overexpression of active GSK3β in murine forebrains was associated with tau hyperphosphorylation and somatodendritic tau accumulation in hippocampal neurons [160]. Interestingly, tau-deficient mice overexpressing GSK3β displayed reduced neurodegenerative symptoms and milder cognitive deficits, suggesting that the interaction between GSK3β and tau is critical for tau hyperphosphorylation and the development of AD [161]. Furthermore, postmortem examinations of brains of AD patients showed elevated levels of GSK3β in comparison to non-AD patients of the same age [162].

GSK3β has been suggested to connect Aβ and tau in the pathogenesis of AD [163]. Aβ formation has been observed to result in the activation of GSK3β by activating RhoA/ROCK through the phosphorylation of the Tyr42 residue in RhoA [164]. Once activated, RhoA/ROCK is thought to activate Src, which then phosphorylates GSK3β at the Tyr216 residue [164]. The linking pathway between the presence of Aβ and activated RhoA/ROCK is not fully understood, but it is thought that Aβ oligomers bind to the p75 neurotrophin receptor (p75^NTR^) [165]. Paradoxically, GSK3β has also been shown to regulate the production of Aβ [166,167]. Aβ is formed from successive proteolytic cleavages of APP. APP is a transmembrane protein abundantly expressed in the brain and is subject to two distinct metabolic pathways regulated by secretases [168]. The nonamyloidogenic pathway is modulated by α-secretases that cleave APP into easily degradable fragments [169]. Conversely, the amyloidogenic pathway is mediated by a β-secretase (BACE-1) and γ-secretase complex. The amyloidogenic cleaving of APP forms an Aβ peptide that aggregates in AD brains [170]. GSK3β regulates Aβ production by phosphorylating and mediating the activation of presenilin-1 (PS1), a component of the γ–secretase complex [167]. Alternatively, GSK3β, through NF-κB overexpression, is thought to upregulate BACE-1 expression [171]. While the upregulation of BACE-1 by GSK3β increases Aβ cleavage, the effects of GSK3β phosphorylation of PS1 and the resulting modulation of γ–secretase activation on Aβ processing are unclear and are a necessary area for future investigation [159,172]. Finally, it is suggested that Aβ blocks Wnt signaling-induced GSK3β deactivation, leading to increased GSK3β activation. It is thought that Aβ can bind to the extracellular domain of the Wnt ligand-receptor Frizzled (FZD), disrupting the interactions between Wnt ligands and the Low-Density Lipoprotein Receptor-Related Protein 6 (LRP6)/Fz complex [173]. Increased GSK3β activation results in an increase in Aβ cleavage and tau hyperphosphorylation, completing the positive feedback loop [173]. This positive feedback loop underscores the central role of GSK3β as a critical mediator linking Aβ production, tau hyperphosphorylation, and the amplification of pathological signaling in AD (Figure 2).

GSK3β-induced tau hyperphosphorylation impairs axonal transport, leading to further cognitive decline [174]. Axonal transport disturbances are an early hallmark of neurological disorders including AD [174]. While GSK3β is required to phosphorylate kinesin-1 to induce axonal transport of tau, the hyperphosphorylated tau disrupts axonal trafficking by destabilizing the MT cytoskeleton, diminishing the binding between motor proteins and cargo [175]. It is suspected that hyperphosphorylated tau impairs the function of c-Jun N-terminal kinase-interacting protein 1 (JIP1), which is responsible for facilitating the binding of cargo to motor proteins, although the complete mechanism is not clear [176]. Loss of axonal transport leads to vesicular aggregation and subcellular mislocation [177,178]. Additionally, the overactivation of GSK3β is thought to decrease cholinergic function, parallel to the reductions in cholinergic neurons exhibited in AD brains [179,180]. GSK3β is able to inhibit the production of the cholinergic neurotransmitter acetylcholine (ACh) by phosphorylating and deactivating pyruvate dehydrogenase, an essential enzyme for the functioning of choline acetyltransferase (ChAT), which is responsible for synthesizing ACh [179,181]. The loss of ACh causes the loss of function in cholinergic neurons, inducing further cognitive impairment [180].

GSK3β also plays an essential role in modulating cognitive functions at the presynaptic and postsynaptic levels. Within presynaptic regions, the overactivation of GSK3β hinders the exocytosis of synaptic vesicles by phosphorylating P/Q-type calcium channels and disrupting the soluble NSF attachment protein receptor (SNARE) complex formation [182]. Such inhibition significantly diminishes the presynaptic release of glutamate and the clustering of synapsin I, a protein crucial for the release of neurotransmitters, preventing synapse formation [183]. Contrastingly, at the postsynaptic level, GSK3β regulates synaptic plasticity by mediating long-term potentiation (LTP) and long-term depression (LTD) [184,185,186]. It has been shown that LTP induction inhibits GSK3β activation by an increase in phosphorylated Ser9 levels, while transgenic mice overexpressing active GSK3β showed impaired LTP, suggesting that overactive GSK3β may impair LTP in cognition-associated conditions [183,187,188]. Further, is it suggested that GSK3β activation facilitates the induction of N-methyl-D-aspartate receptor (NMDAR)-mediated LTD through mechanisms involving PP1-mediated dephosphorylation and Akt inhibition downstream of NMDAR activation, strongly suggesting that overactivated GSK3β supports the induction of abnormal levels of LTD observed in multiple neuropsychiatric disorders [188,189,190,191,192,193]. It is noteworthy that GSK3β may be involved in the cross-communication between LTP and LTD [186]. Multiple studies have shown that inhibitors of GSK3β may be able to help normalize abnormal levels of both LTP and LDP in the brain, as treatment with GSK3β inhibitors was accompanied by increased cognitive abilities in mouse models with Fragile X syndrome, and Down syndrome, and AD [189,190,191,192,193].

## 6. sTREM2 as a Regulator of RhoA/ROCK/GSK3β Signaling

Multiple studies have suggested that the solubilized form of triggering receptors expressed on myeloid cells 2 (sTREM2) can reduce Aβ accumulation and slow AD progression [119,194,195,196,197]. TREMs are a broadly expressed family of cell surface receptors. TREMs primarily act as modulators of the immune response that regulate the activation of myeloid cells, notably macrophages [198,199]. Within the family, TREM2 is the most widely studied molecule and is implicated in the pathogenesis of numerous macrophage-associated and inflammation-related diseases such as AD [200]. When membrane-bound, TREM2 binds to lipids and elicits essential neuroprotective effects by downregulating the expression of pro-inflammatory cytokines, such as TNF-α, IL-1β, and NOS2, and upregulating the transcription of anti-inflammatory cytokines, including IL-4, IL-10, and IL-11 [201,202,203]. Additionally, TREM2 signaling has been shown to mediate the expression of the activation of Toll-like receptors (TLRs) [204]. In the brain, TREM2 is highly expressed by microglia in the temporal cortex surrounding Aβ plaques [205]. The TREM2 signaling pathway is involved in the microglial response and clearance of amyloid plaques, and is critical for successful synaptic pruning in the brain [206,207]. It has been shown with microglial RNA-seq analysis that TREM2 is required to sustain the microglial response to clear or prevent Aβ plaque formation [208,209].

The ectodomain of TREM2 can be solubilized by the α-secretases disintegrin and metalloproteinase domain-containing protein 10 (ADAM10) and ADAM17 at the His157-Ser158 bond within the stalk region to form soluble TREM2 (sTREM2) [210]. Studies revealed that elevated concentrations of sTREM2 are released by microglia into cerebral spinal fluid (CSF) in AD patients, although the extracellular conditions that increase or decrease sTREM2 shedding are uncertain [211]. sTREM2 has been suggested to influence the pathogenesis of AD, but the mechanisms behind the effects are unclear. Putatively, it is believed that sTREM2 plays a protective role in AD development [212]. This notion was supported by studies that show sTREM2 is able to block the aggregation and neurotoxicity of Aβ plaques in murine models [213,214,215]. Furthermore, it has been suggested that sTREM2 reduces cognitive impairments induced by tau pathologies, such as preventing the loss of hippocampal synapses in tau P301S mice [9,216]. However, one study demonstrated that the injection of sTREM2 into the brains of healthy mice increased levels of pro-inflammatory cytokine production and microglial activation and proliferation [216]. Another study has demonstrated that sTREM2 may act as a decoy receptor and have indirect pro-inflammatory effects by reducing the modulatory anti-inflammatory function of TREM2 signaling [217]. It must be noted that the impact of sTREM2 on the cognitive state is dependent on various factors, including differing genetic variants, biogenesis mechanisms, patient demographics, environmental factors, and pathological contexts [218]. For example, it is suggested that the R47H variant of sTREM2 exhibits a reduced binding frequency and affinity to Aβ in comparison to wild-type sTREM2. Consequently, WT sTREM2 disaggregates protofibrils and larger Aβ oligomers into the smallest Aβ oligomers, effectively inhibiting Aβ aggregation. In contrast, the R47H mutant was found to promote the formation of Aβ protofibrils, partially explaining its association with an increased risk for AD [213]. Although sTREM2 is generally considered advantageous in mitigating AD pathogenesis, it may also promote microglial activation and neuroinflammation, which may aggravate neurodegeneration in specific contexts [218]. Thus, the underlying mechanisms behind its neuroprotective effects are a crucial area for future investigations before sTREM2 can be explored as a therapeutic avenue.

Neuronal Transgelin-2 (TG2 or SM22β), an actin-binding protein, has recently been suggested to ligate sTREM2 and deactivate the RhoA/ROCK/GSK3β signaling pathway, which may explain the neuroprotective effects observed following the shedding of sTREM2 [9]. TG2 is encoded by the *TAGLN2* gene and is one of three transgelin isoforms alongside TG1 and TG3 [219]. Transgelins characteristically have transformation and conformation-sensitive properties, although the full implications of such are not fully known [220]. TG2 is highly expressed in smooth muscle cells [221]. It is also found in non-smooth muscle cells, including fibroblasts, epithelial cells, and immune cells throughout the body [222,223,224]. In the brain, TG2 is expressed primarily in neurons and microglia [9,225]. Broadly, TG2 is involved in regulating actin binding and stabilization, smooth muscle contraction, cell motility, and migration [226,227,228].

Recently, an interesting study by Zhang et al., 2023, proposed that TG2 expressed on neurons may serve as a receptor for sTREM2 [9]. Affinity purification pulldown and affinity purification mass spectrometry revealed sTREM2 and TG2 colocalized on the cell surface of hippocampal neurons from AD patients and tau P301S transgenic mice, implying that TG2 serves as a sTREM2 receptor [9]. In support of this, TG2 has recently been shown to localize at the cellular membrane and function as a regulatory receptor of the myosin cytoskeleton and airway smooth muscle [221]. It has been shown that the TG2 agonist, TSG12, induced RhoA phosphorylation at the Ser188 residue, deactivating the molecule and, by extension, inhibiting RhoA/ROCK/GSK3β signaling [164,221]. Furthermore, neuroblastoma cell lines treated with sTREM2 exhibited decreased levels of activated RhoA and elevated levels of inactive RhoA phosphorylated at the Ser188 residue [9,229,230]. Further treatment with RhoA and ROCK inhibitors, Tat-C3 and Y-27632, respectively, emulated the inhibitory effect of sTREM2 on GSK3β activation and subsequent tau phosphorylation [9]. Additional transfection of phosphorylation-resistant RhoA (RhoA S188A) into an endogenous RhoA-null background silenced the inhibitory effect of sTREM2 [9]. It was observed that sTREM2 dramatically reduced the phosphorylation of tau at S202 and S396 residues, identical sites that are targets of GSK3β phosphorylation [9,231]. Concentration-dependent sTREM2 was also observed to significantly decrease the phosphorylation of GSK3β at the Tyr216 residue, and inhibition of sTREM2 with an anti-TREM2 antibody depleted this effect, suggesting that sTREM2 is able to deactivate GSK3β through TG2 ligation [9,72]. Finally, TG2 knockdown attenuated the neuroprotective function of sTREM2 on the inhibitory and excitatory synapses in the hippocampus of P301S mice [9]. This study strongly suggests that the ligation of TG2 by sTREM2 deactivates the RhoA/ROCK pathway through inhibiting RhoA, effectively preventing GSK3β activation and successive tau hyperphosphorylation. While the data from this study provide solid evidence proving the link between sTREM2 and tau phosphorylation and AD behavioral defects, whether this pathway impacts AD progression warrants further investigation using clinically relevant mouse models.

**Figure 3:**
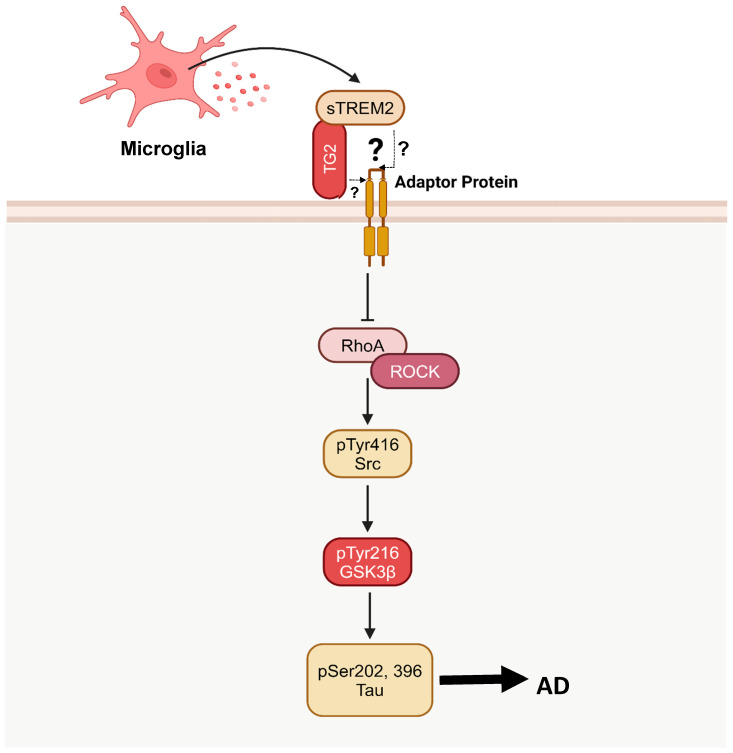
A signaling pathway depicting the potential for TG2 to serve as a sTREM2 receptor and inhibit the RhoA/ROCK/GSK3β pathway. A potential adaptor molecule is shown, as TG2 lacks a transmembrane domain. However, what this adaptor protein is, and if there truly is an adaptor protein altogether, is still under investigation.

Although TG2 was suggested to act as a cell surface receptor, it is primarily a cytosolic protein and is not known to possess a transmembrane domain when localized at the cellular membrane [232,233]. Thus, TG2 is not capable of inducing or inhibiting a signaling cascade directly. Alternatively, it is possible that TG2 may be coupled in low affinity with an adaptor molecule in the transmembrane domain that would not be detected in an affinity pulldown assay, potentially explaining how the ligation of TG2 exhibits RhoA/ROCK inhibition (Figure 3). Perhaps TG2 exhibits transient, low-affinity interactions with lipid raft-resident adaptor proteins or actin-linked scaffolding molecules. Given the proximity of TG2 to cortical actin networks, it is possible that TG2 interacts with adaptor proteins via weak electrostatic forces or PTMs that mediate binding affinity [9]. This hypothesis can be verified through a co-immunoprecipitation assay. Considering the potential low-affinity binding, a cross-linker should be utilized. Notwithstanding this, the interaction between sTREM2, TG2, and the RhoA/ROCK pathway warrants further investigation.

## 7. Emerging Perspectives and Challenges

Given the pivotal role of GSK3β overactivation in tau hyperphosphorylation, many preclinical studies attempted to inhibit GSK3β signaling as a therapeutic target for AD. Inhibitors including 6-bromoindirubin-3’oxime (6BIO), Hymenialdisine (HD), CHIR98014, SB-216763, and Alsterpaullone have been tested in preclinical studies in mouse models, with decreased tau phosphorylation, Aβ deposition, inflammation, and spatial memory deficits [234,235,236,237,238]. Preclinical studies have also suggested potential offsite toxicity due to suboptimal GSK3β specificity [235]. Targeting GSK3β has advanced to clinical trials (Table 1); while some results are encouraging, they are mostly inconclusive and unsatisfactory at this moment.

Although the therapeutic targeting of GSK3β is promising, it is still in its infancy, pending the resolution of difficulties, most notably specificity. Due to the broad expression of GSK3β and numerous physiological processes throughout the body, such as cell growth and survival, the inhibition of GSK3β in general will likely lead to adverse effects. Moreover, due to the broadly conserved ATP-binding sites across the kinase family, engineering a small molecule that can effectively target specific kinases is exceedingly challenging. Nevertheless, developing a selective inhibitor to specifically target hyperactive GSK3β in neurons may avoid such adverse effects. However, the difficulty in developing a GSK3β-specific inhibitor is further solidified by blood–brain barrier (BBB) penetration, as an inhibitor must be small enough to pass through the BBB. A cargo such as a nanoparticle may be considered.

Given the aforementioned challenges of targeting GSK3β in treating AD, targeting upstream pathways leading to GSK3β activation in neurons may be offer new treatment avenues. The intricate signaling network of RhoA/ROCK/GSK3β establishes it as a significant and promising immunotherapeutic target for treating AD. The immensely wide evolutionary conservation of GSK3β suggests that the *GSK3β* gene is essential for regulating synaptic function. Dysregulation of this pathway has been shown to largely contribute to tau hyperphosphorylation, neuroinflammation, and synaptic dysfunction, leading to cognitive decline. While targeting the molecules of this pathway for therapeutic treatment of AD appears to be propitious, much remains to be understood about the complex functions and signaling cascades involved, especially in the context of disease progression. The RhoA/ROCK/GSK3β pathway intersects with multiple cellular processes, and its modulatory functions have been shown to be highly context-dependent. Future research is imperative to fully understand the mechanisms behind this pathway and its influence on AD.

While sTREM2 has been suggested to be a promising immunotherapeutic target, the participation of sTREM2 in AD pathogenesis seems highly complex, based on many controversial findings. How sTREM2 plays a role in AD pathogenesis must be fully understood before therapeutic interventions. Given that TG2 has recently been demonstrated to have a positive role in reducing AD development [9], strategies to increase TG2 expression in neurons may be a viable therapeutic angle.

## Figures and Tables

**Table 1:** Clinical Trials Targeting GSK3β Signaling.

NCT/PMID	Drug Name	Mechanism of Action	Outcome	Phase
NCT02129348	Lithium Carbonate	Reversible small molecule GSK3 inhibitor	- Increased aggression - Moderate/marked improvement - Improvement on NPI delusions and irritability	Phase II
NCT00088387	Lithium Carbonate & Divalproex	- Co-inhibition of GSK3 - Histone deacetylase (HDAC) inhibition - Sodium channel modulation	No reported results	Phase II
NCT00948259	Tideglusib	Irreversible small molecule GSK3 inhibitor	No reported results	Phase I, Phase II
NCT01055392	Lithium Carbonate	Reversible small molecule GSK3 inhibitor	- Improved performance on memory and attention tests for 24 months - Significant increase in CSF Aβ after 36 months - Increased adverse events	Phase II
